# Panel-based testing for inherited colorectal cancer: a descriptive study of clinical testing performed by a US laboratory

**DOI:** 10.1111/cge.12359

**Published:** 2014-03-20

**Authors:** D Cragun, C Radford, JS Dolinsky, M Caldwell, E Chao, T Pal

**Affiliations:** a H. Lee Moffitt Cancer CenterTampa, FL, USA; b Ambry GeneticsAliso Viejo, CA, USA; c Department of Pediatrics, University of CaliforniaIrvine, CA, USA

**Keywords:** clinical genetics, ColoNext, hereditary cancer syndromes, multiplex genetic testing, next generation sequencing, variants of unknown significance

## Abstract

Next-generation sequencing enables testing for multiple genes simultaneously (‘panel-based testing’) as opposed to sequential testing for one inherited condition at a time (‘syndrome-based testing’). This study presents results from patients who underwent hereditary colorectal cancer (CRC) panel-based testing (‘ColoNext™’). De-identified data from a clinical testing laboratory were used to calculate (1) frequencies for patient demographic, clinical, and family history variables and (2) rates of pathogenic mutations and variants of uncertain significance (VUS). The proportion of individuals with a pathogenic mutation who met national syndrome-based testing criteria was also determined. Of 586 patients, a pathogenic mutation was identified in 10.4%, while 20.1% had at least one VUS. After removing eight patients with *CHEK2* mutations and 11 *MUTYH* heterozygotes, the percentage of patients with ‘actionable’ mutations that would clearly alter cancer screening recommendations per national guidelines decreased to 7.2%. Of 42 patients with an ‘actionable’ result, 30 (71%) clearly met established syndrome-based testing guidelines. This descriptive study is among the first to report on a large clinical series of patients undergoing panel-based testing for inherited CRC. Results are discussed in the context of benefits and concerns that have been raised about panel-based testing implementation.

Conflict of interest

Cristi Radford and Jill Dolinsky are full-time employees for the commercial laboratory Ambry Genetics, which performs ColoNext™ testing. Elizabeth Chao is a paid consultant for Ambry. Deborah Cragun, Meghan Caldwell, and Tuya Pal report no potential conflicts of interest. Specifically, they are not employed by Ambry, and they did not receive any financial or other incentives from Ambry.

Colorectal cancer (CRC) is the second leading cause of cancer-related death in the United States when men and women are considered together 1. The estimated fraction of CRC attributed to inherited predisposition ranges between 10% and 30% 2. Identification of hereditary CRC syndromes can lead to reductions in morbidity and mortality through targeted risk management options 3,4. Concurrently, advances in DNA sequencing technologies (called next-generation sequencing; NGS) now make it possible to test for multiple genes simultaneously (panel-based testing), at a cost comparable to testing for two genes using older methods (syndrome-based testing) 5. As a result, it is possible that the conventional syndrome-based approach to performing inherited cancer predisposition testing, which includes generating a differential diagnosis and sequentially testing for single genetic conditions, may shift to panel-based tests.

In March 2012, Ambry Genetics Corporation (Ambry; Aliso Viejo, CA) was the first clinical laboratory to offer hereditary cancer panel-based testing in the United States; subsequently, other laboratories have started offering cancer panels. The 14 genes included in Ambry's colon panel-based testing (ColoNext™) are listed in Table1 along with their associated cancer risks. Although hereditary cancer panels vary, they typically include both highly penetrant as well as moderately penetrant genes 6. For highly penetrant genes, clinical guidelines exist for the prevention or early detection of cancers 7. In other words, these are ‘actionable genes’ with known clinical utility 8. In contrast, the utility of moderately penetrant genes is less certain.

**Table 1:** Genes included in ColoNext, associated cancer risks, and established syndrome-based testing criteria

Cancer syndrome^a^	Gene(s)	Associated cancer sites (risks)^b^	Actionable^c^ yes/no cancer type	Testing/screening/diagnostic criteria^d^
Lynch syndrome (hereditary non-polyposis colorectal cancer; HNPCC)	*MLH1* *MSH2* *MSH6* *PMS2* *EPCAM*	Cancer site (risk up to age 70) Colon (40–80% *MLH1, MSH2*) (10–22% *MSH6, PMS2*) Endometrium (25–60% *MLH1, MSH2*) (15–26% *MSH6, PMS2*) Increased cancer risks: stomach, ovarian, hepatobilliary tract, urinary tract, small bowel, others	Yes Colon Endometrial Ovarian Urothelial	*LS tumor screening* recommended for all patients with CRC or CRC patients <70 and those ≥ 70 meeting Revised Bethesda Criteria for testing^e^ or *Amsterdam II criteria* (diagnostic of HNPCC if all of following are present): Three or more relatives with an associated cancer (colorectal cancer, or cancer of the endometrium, small intestine, ureter or renal pelvis) Two or more successive generations affected One or more relatives diagnosed before the age of 50 years One should be a first-degree relative of the other two Familial adenomatous polyposis (FAP) should be excluded in cases of colorectal carcinoma Tumors should be verified by pathologic examination
Juvenile polyposis syndrome (JPS)	*BMPR1* *SMAD4*	Cancer site (lifetime risk) Colon (40–50%) Stomach (21% if multiple polyps) Small intestine (rare) Pancreas (rare)	Yes Colon Stomach	Diagnostic criteria^f^ At least 3–5 juvenile polyps of the colorectum or Multiple juvenile polyps throughout the GI tract or Any number of juvenile polyps and family history of juvenile polyps
Peutz–Jeghers (PJS)	*STK11*	Cancer site (lifetime risk) Breast (45–50%) Colon (39%) Stomach (29%) Pancreas (11–36%) Small intestine (13%) Ovary (18–21%) Cervix, uterus, testes, lung	Yes Breast Colon Stomach Pancreas Small intestine Ovary, cervix, uterus, testes	Individual with multiple GI hamartomatous polyps
Familial adenomatous polyposis (classic FAP) and attenuated FAP (AFAP)	*APC*	Colon (nearly 100% FAP) (70% AFAP) Duodenal (4–12%) Hepatoblastoma (1–2% by age 5) Thyroid (<2%)	Yes Colon Duodenal Gastric Thyroid Small bowel Hepatoblastoma	Personal history of >10 adenomas or Personal history of a desmoid tumor
MUTYH-associated polyposis (MAP)	*MUTYH* (biallelic mutations)	Colon (63% risk up to age 60) 20 Duodenum (occurs in 5% of patients NCCN)	Yes Colon Duodenal	Personal history of >10 adenomas or ≥ 5 serrated polyps with at least some adenomas
No associated syndrome	*CHEK2*	Moderate increased risk of developing many types of cancer including breast, prostate, colon, thyroid, ovarian, and kidney (19)	No Consensus for optimal management or surveillance has not been reached^g^	No defined criteria as this is a moderate penetrance gene
Li–Fraumeni and Li–Fraumeni like syndrome (LFS)	*TP53*	Bone and soft tissue sarcomas Premenopausal breast cancer Brain tumors (astrocytoma, glioblastoma, medulloblastoma Choroid plexus carcinoma) Adrenocortical carcinoma Colon cancer Acute leukemia 60% risk of developing associated cancers by age 45; 95% by age 70	Yes Breast Colon Other screening or surveillance approaches are under investigation	*Classic LFS if all of the following*: Proband with sarcoma diagnosed before age 45 years A first-degree relative with cancer before age 45 A first- or second-degree relative with any cancer before age 45 years or a sarcoma at any age or *Chompret criteria any ONE of the following*: Proband with a tumor belonging to the LFS tumor spectrum before age 46 years AND at least one first- or second-degree relative with a LFS tumor (except breast cancer if the proband has breast cancer) before age 56 years or with multiple tumors Proband with multiple tumors (except multiple breast tumors), two of which belong to the LFS tumor spectrum and the first of which occurred before age 46 years Proband with adrenocortical carcinoma or choroid plexus tumor, regardless of family history or *Early-onset breast cancer*: Individual with breast cancer ≤35 y with negative *BRCA* test
PTEN Hammartoma tumor syndrome (PHTS); Cowden syndrome; Bannayan–Riley–Ruvalcaba syndrome	*PTEN*	Breast (75–85% lifetime) Thyroid (3–35% lifetime) Endomentrium (19–28%) Colon (9–16%) Renal (15–34%)	Yes Breast Thyroid Colon	*Individual with personal history of one of the following*: Bannayan–Riley–Ruvalcaba syndrome Adult Lhermitte–Duclos disease Autism spectrum disorder and macrocephaly Two or more biopsy-proven trichilemmomas Two or more major criteria (one must be macrocephaly) Three major criteria, without macrocephaly One major and ≥ three minor criteria ≥Four minor criteria *Major criteria* Breast cancer Endometrial cancer Follicular thyroid cancer Multiple GI hamartomas or ganglioneuromas Macrocephaly (≥97th percentile) Macular pigmentation of glans penis
				Specified mucocutaneous lesions (trichilemmoma, palmoplantar keratosis, oral mucosal papillomatosis, facial papules) *Minor criteria* Autism spectrum disorder Colon cancer Esophageal glycogenic acanthosis (≥3) Intellectual disability (IQ ≤75) Papillary or follicular variant of papillary thyroid cancer Other thyroid lesions (e.g., adenoma, goiter) Renal cell carcinoma Single GI hamartoma or ganglioneuroma Lipomas Testicular lipomatosis Vascular anomalies (multiple intracranial developmental venous anomalies)
Hereditary diffuse gastric cancer (HDGC)	*CDH1*	Cancer site (lifetime risk) Gastric cancer (67–83%) Lobular breast cancer (39–52%) CRC (risk undefined)	Yes Gastric^h^ Breast No Colon	Testing criteria per consortium consensus guideline (Fitzgerald, et. al, 2010) Family with ≥2 cases of diffuse gastric cancer (DGC), with at least one DGC diagnosed <age 50 or Three first or second degree relatives with DGC independent of age or Personal or family history of DGC and lobular breast cancer, one dx <50 or Single individual with DGC dx before age 40 years

MAP, *MUTYH* associated polyposis; NCCN, National Cancer Center Network; DGC, diffuse gastric cancer; HDGC, hereditary diffuse gastric cancer.

a All of the cancer syndromes, except MAP, are autosomal dominant with variable penetrance and expressivity. MAP is autosomal recessive. *CHEK2* is not associated with a syndrome, but moderately increased cancer risks occur among individuals with a single deleterious mutation.

b All risks are based on information in the 2013 National Cancer Center Network (NCCN) guidelines.

c HDGC is defined as ‘actionable’ based on a published consensus statement (28). For all other conditions, ‘actionable’ is defined according to whether there are associated NCCN recommended screening/prevention guidelines (NCCN, 2013) 7.

d Testing criteria are based on 2013 NCCN guidelines unless otherwise specified.

e Revised Bethesda Criteria were proposed as a method for selecting which tumors should be screened to help identify patients who should be offered germline testing for Lynch syndrome (29).

f No testing criteria was available in NCCN guidelines, therefore NCCN diagnostic criteria are listed instead.

g Screening recommendations are based on family history, though a *CHEK2* mutation may influence recommendations.

As panel-based testing is implemented into clinical practice, cost-saving opportunities need to be considered. In particular, the most common cause of hereditary CRC is Lynch syndrome, which is caused by mutations in five genes (i.e. *MLH1, MSH2, MSH6, PMS2, EPCAM*). When this study was initiated, syndrome-based testing for the Lynch syndrome genes through Ambry cost $100 more than ColoNext™. However, pricing has fluctuated and ColoNext™ testing has become more expensive than testing for the five Lynch syndrome genes. Cost analyses are further complicated because tumor screening for Lynch syndrome using immunohistochemical (IHC) testing may narrow down the number of genes that require testing 9.

Despite the potential for panel-based testing to identify more mutations, this testing is also expected to increase the complexity of results interpretation because of factors such as questionable or uncertain clinical utility of testing for moderate penetrance genes and the higher rate of inconclusive results because of an increase in the number of variants of uncertain significance (VUS) 6,10,11. Furthermore, given that widespread panel-based testing for hereditary CRC only began in 2012, little is known about patients who are tested or rates of identified mutations and variants. To address these questions, we examined a comprehensive data repository of panel-based testing for inherited susceptibility to CRC that is maintained through Ambry. Unlike prior validation studies of cancer panel-based tests 5,12, the purpose of this study was to describe the clinical use and results of ColoNext™ testing performed on a clinical basis for diagnostic purposes in patients without previously identified mutations. The aims of this study were to estimate mutation and VUS rates identified through panel-based testing for hereditary CRC in real-world settings and to determine whether patients with a mutation met national genetic testing criteria for the respective cancer syndromes identified.

## Materials and methods

### Data source

A database maintained by Ambry includes demographic information as well as personal and family cancer history collected from ordering clinicians via test requisition forms (TRF). Data extracted by Ambry for use in this study included de-identified information on individuals for whom ColoNext™ testing was completed between March 2012 (when ColoNext™ became available) and March 2013. Following receipt of an exempt certification by the University of South Florida's institutional review board, the first and senior authors (D. C. and T. P.) conducted secondary data analysis on the de-identified dataset.

### ColoNext™ testing and results reporting

All genetic testing was performed by Ambry using the following protocol. Genomic deoxyribonucleic acid (gDNA) was isolated from patients' whole blood specimens, or from saliva specimens collected using an Oragene kit. Sequence enrichment of each coding exon within the 14 genes was carried out by incorporating the gDNA into microdroplets along with primer pairs designed to the specified target. The enriched libraries were then applied to the solid surface flow cell for clonal amplification and sequencing using paired-end, 100-cycle chemistry on the Illumina Hiseq 2000 (Illumina, San Diego, CA). For 13 of the 14 cancer susceptibility genes, NGS/Sanger sequencing was performed for all coding domains plus at least five bases into the 5′ and 3′ ends of all introns and untranslated regions. Sequence analysis was not performed for *EPCAM*, as currently the only mutations in *EPCAM* associated with Lynch syndrome are gross deletions encompassing its 3′ terminus 13. Additional Sanger sequencing was performed for any regions with insufficient depth of coverage by NGS, with an initial read depth threshold of at least 10 times and a quality score of 20 or better. This threshold was later increased to a read depth of 50 times with a quality score of 20 or better. Variant calls other than known, non-pathogenic alterations were verified by Sanger sequencing in sense and antisense directions prior to reporting. A targeted chromosomal microarray (CMA) designed with increased probe density in regions of interest was used for the detection of gross deletions and duplications for each sample (Aglient, Santa Clara, CA). Owing to potential pseudogene interference 14, *PMS2* sequence analysis was performed via long range PCR followed by Sanger sequencing and *PMS2* deletion/duplication analysis was performed via MLPA. If a gross deletion was detected in exons 12, 13, 14, or 15 of *PMS2*, double stranded sequencing of the appropriate exon(s) of the pseudogene was performed to determine if the deletion was located in *PMS2* or the pseudogene. Alterations were classified based on guidelines from the International Agency for Research on Cancer (IARC) 15 and the American College of Medical Genetics (ACMG) 16 into the following categories: (1) pathogenic mutation; (2) variant, likely pathogenic; (3) variant, unknown significance; (4) variant, likely benign; (5) benign.

### Data analysis

Those patients with more than one gene alteration were categorized according to the most severe result as follows: (1) positive (mutation), pathogenic mutation or likely pathogenic variant (regardless of whether other variants were identified); (2) variant of unknown significance (VUS); (3) variant, likely benign (VLB); (4) negative, no mutations or variants. Those with a positive result were further sub-divided according to whether the mutation was actionable indicating that it would alter cancer screening recommendations per guidelines from the National Comprehensive Cancer Network (NCCN) 7. Mutation detection rates were calculated for both positive and actionable results. Additionally, the proportion of individuals with a VUS was calculated.

Personal and family history information was manually reviewed and coded according to the presence or absence of colon cancer, endometrial cancer, other cancers, and colon polyps. Ordering provider information was coded to reflect involvement of a genetic professional (i.e. board certified/eligible medical geneticist or genetic counselor) for cases meeting one of the following criteria: (1) a master's trained genetic counselor or medical geneticist was listed on the TRF; or (2) in cases where no genetic professional was listed, a search of Ambry's internal database of providers and publically available websites revealed that the ordering provider works closely with a genetic counselor. In order to characterize the population of patients who underwent testing, frequencies for available demographic, clinical, family history, and healthcare provider variables were calculated. Frequencies were also calculated after sub-grouping according to a positive test result, and further sub-dividing according to whether the positive result was actionable.

Personal and family history information for all patients with an actionable result was reviewed by the first author (D. C.) and independently verified by at least one of the other study co-authors (T. P. or J. D.) to determine whether individuals met the respective NCCN syndrome-based testing guidelines presented in Table1. Finally, available clinical and family history information was summarized for all patients with a *CHEK2* mutation.

## Results

### Demographic and clinical characteristics

Our series consisted of 586 individuals who underwent ColoNext™ testing between March 2012 and March 2013. Testing was ordered at 216 unique institutions across the US. The majority of individuals tested were female (60%) and White (72%). Just over half (*n* = 311; 53%) had a personal history of CRC (with or without a history of other cancers or polyps), 105 (18%) had a history of cancer other than CRC, and 123 (21%) had a history of polyps with no personal cancer history. The majority (*n* = 316; 54%) had a positive family history of CRC/or other cancers. Most tests (89%) were ordered by genetic professionals or physicians who work closely with a genetic counselor. Provider settings were diverse, including private offices, community hospitals, and academic institutions. When sub-dividing by test result (i.e. positive and actionable), demographic and clinical characteristics remained similar across the groups (Table2).

**Table 2:** Demographic characteristics of all patients who received ColoNext testing, sub-divided based on the presence of a pathogenic or likely pathogenic result, and further subdivided according to whether the result is ‘actionable’

		Pathogenic or likely pathogenic		Consensus exists on screening/prevention^a^ (i.e. actionable)	
Variable	All patients (*n* = 586)	No (*n* = 525)	Yes (*n* = 61)	No (*n* = 19)	Yes (*n* = 42)
Gender (% female)	354 (60.4)	318 (60.6)	36 (59.0)	14 (73.7)	22 (52.4)
*Ethnicity*					
Caucasian	422 (72)	374 (71.2)	48 (78.7)	18 (94.7)	30 (71.4)
African American (black)	27 (4.6)	26 (5.0)	1 (1.6)	0 (0)	1 (2.4)
Hispanic	30 (5.1)	28 (5.3)	2 (3.3)	0 (0)	2 (4.8)
Jewish	25 (4.4)	23 (4.4)	3 (4.9)	0 (0)	3 (7.1)
Asian	6 (1.0)	6 (1.1)	0 (0)	0 (0)	0 (0)
Mixed ethnicity	12 (2.0)	11 (2.1)	1 (1.6)	0 (0)	1 (2.4)
Other	11 (1.9)	10 (1.9)	1 (1.6)	0 (0)	1 (2.4)
Not specified	52 (8.9)	47 (9.0)	5 (8.2)	1 (5.3)	4 (9.5)
*Billing*					
Insurance	546 (93.2)	488 (93.0)	58 (95.1)	19 (100)	39 (92.9)
Institutional	34 (5.8)	32 (6.1)	2 (3.3)	0 (0)	2 (4.8)
Self-pay	4 (0.7)	3 (0.6)	1 (1.6)	0 (0)	1 (2.4)
Other	2 (0.3)	2 (0.4)	0 (0)	0 (0)	0 (0)
*Ordering provider*					
Genetic counselor (GC) or geneticist listed on TRF	261 (44.5)	233 (44.4)	28 (45.9)	7 (36.8)	21 (50.0)
MD works with GC not listed on TRF	280 (47.8)	252 (48.0)	28 (45.9)	11 (57.9)	17 (40.5)
Other non-genetics provider	45 (7.7)	40 (7.6)	5 (8.2)	1 (5.3)	4 (9.5)
*Healthcare setting*					
University medical center	92 (14.7)	86 (16.4)	6 (9.8)	2 (10.5)	4 (9.5)
Non-university medical center	28 (4.8)	24 (4.6)	4 (6.6)	1 (5.3)	3 (7.1)
Hospital	159 (27.1)	144 (27.4)	15 (24.6)	5 (25.3)	10 (23.8)
Outpatient clinic/private office	261 (44.5)	245 (46.6)	33 (54.1)	11 (57.9)	22 (52.4)
Other	29 (4.9)	26 (5.0)	3 (4.9)	0 (0)	3 (7.2)
Personal hx colon cancer and other cancer (% yes)	70 (11.9)	62 (11.8)	8 (13.1)	1 (5.3)	7 (16.7)
Personal hx colon cancer only (% yes)	242 (41.3)	218 (41.5)	24 (39.3)	6 (31.6)	18 (42.9)
Personal hx other cancer only (% yes)	107 (18.3)	94 (18.1)	13 (21.3)	5 (25.3)	8 (19.0)
Personal hx polyps, but no cancer (% yes)	122 (20.8)	109 (20.8)	13 (21.3)	6 (31.6)	7 (16.7)
Family hx colon cancer first–third degree (% yes)	335 (57.2)	300 (57.1)	35 (57.4)	13 (68.4)	22 (52.4)
Family hx other cancer (% yes)	325 (55.5)	292 (55.6)	33 (54.1)	13 (68.4)	20 (47.6)
Family hx polyps (% yes)	102 (17.4)	95 (18.1)	7 (11.5)	4 (21.1)	3 (7.1)

TRF, test requisition forms; NCCN, National Comprehensive Cancer Network; MD, medical doctor (physician).

a Pathogenic mutations in *CHEK2* are ‘not actionable’ because of lack of evidence and lack of consensus guidelines. Heterozygous pathogenic mutations in *MUTYH* are ‘not actionable’ because *MUTYH* associated polyposis is recessive and requires the person to have biallelic mutations to be at high risk for cancer. Pathogenic mutations in *CDH1* are ‘actionable’ based on a published consensus statement for hereditary diffuse gastric cancer (Fitzgerald et al., 2010). Pathogenic mutations in the other genes on the ColoNext panel are ‘actionable’ because there are NCCN recommended cancer screening or prevention guidelines for the associated conditions.

### Test results

#### Pathogenic and actionable mutations

**Fig 1:**
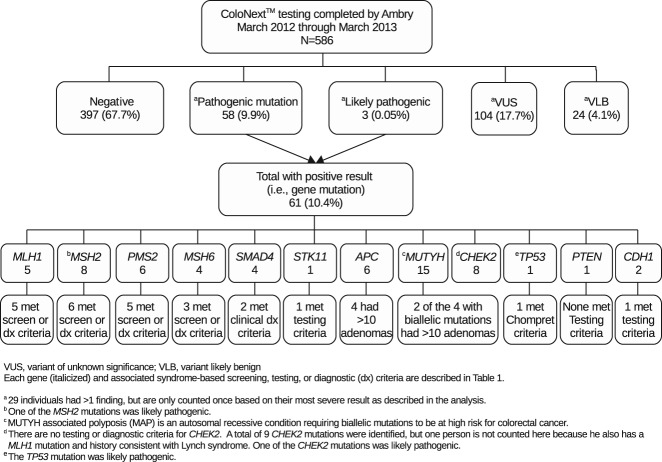
Gene alterations identified through ColoNext™ testing and number of patients with actionable mutations who met NCCN testing, screening, or diagnostic criteria.

Results of ColoNext™ testing are summarized in Fig. 1. Of 586 patients, 61 (10.4%) were positive for a mutation in at least one of the genes analyzed. Only one person had two pathogenic mutations (*MLH1* and *CHEK2*). After removing the 8 other patients with *CHEK2* mutations and all 11 patients in whom only 1 *MUTYH* mutation was identified, the number of patients with actionable mutations decreased to 42 (7.2%), with over half of these occurring in Lynch syndrome genes.

#### Variants of unknown significance (VUS)

A total of 118 individuals (20.1%) had at least one VUS identified. These included: 14 with a pathogenic mutation and one or more VUS; 99 with one VUS; and 15 with more than one VUS (Fig. 1). Among all 159 VUS results, 77 (48%) occurred in one of the genes associated with Lynch syndrome.

#### Syndrome-based testing/screening guidelines

As shown in Fig. 1, of the 42 patients with an actionable mutation, 30 (71%) clearly met NCCN syndrome-based testing, screening, or diagnostic criteria listed in Table1. Available information for those 12 not clearly meeting criteria is included in Table3.

**Table 3:** General descriptions of individuals with a positive ‘actionable’ mutation^a^ who did not clearly meet the testing criteria listed in Table 1

Gender	Gene and mutation	Personal history and age at diagnosis	Family history and age at diagnosis
F	*MSH2* p.A636P	No personal history of cancer	Father – sarcoma, age 63 Paternal grandmother – CRC, age 60; uterine cancer, age 45 Paternal aunt – uterine cancer, age 45
F	*MSH2* p.P349A (likely pathogenic)	Cervical cancer, age 34	Mother – CRC, age 66 Maternal grandmother – stomach cancer, age 82; pancreatic cancer, age 84 Maternal great grandmother – stomach cancer, age 60
F	*PMS2* c.1067delA	Breast cancer, age 55 Cervical dysplasia, age 40s endometrial cancer, age 53 Goiter, age 53	Mother – endometrial cancer, age 65 Maternal uncle – brain tumor, age 75 Maternal uncle – stomach cancer, age unknown Maternal grandmother – CRC, age 56
F	*MSH6* p.Q978X	Endometrial cancer Currently age 70	Mother – CRC, age unknown Maternal aunt – CRC, age unknown Maternal uncle – CRC, age unknown Maternal uncle – pancreatic cancer, age unknown Paternal uncle – CRC, age 72 Paternal uncle – CRC, age unknown
M	*SMAD4* p.Q449X	20–99 adenomatous polyps by age 50 Gastric cancer, age 35 Gastric hyperplastic polyps Colon inflammation Small bowel polyps	Mother – lung cancer 79
F	*SMAD4* c.1245_1248delCAGA	CRC, age 30	Father – melanoma, age 52 Paternal aunt – breast cancer, age 48 Paternal great grandmother – gastric cancer, age unknown Paternal grandfather – lung cancer, age 72
M	*APC* c.2004delC	CRC, age 39 2–5 adenomatous polyps	Maternal uncle – prostate cancer, age unknown Maternal uncle – prostate cancer, age unknown
F	*APC* c.3260_3261delTC	CRC, age 29 Medulloblastoma, age 12	Father – colon cancer, age 46
F	*MUTYH* p.Y179C & p.G396D	CRC, age 41 Endometrial cancer, age 51 Prior Lynch testing negative	Maternal Grandmother – breast cancer, ages 60 and 90 Paternal aunt – breast cancer, age 40's Brother – CRC, age 55; thyroid cancer, age 55 Maternal Uncle – prostate cancer, age 65
F	*MUTYH* p.Y179C & p.G396D	2–5 adenomatous polyps, age 26 CRC, age 26	Father – 6–7 polyps, age 54 Paternal grandmother – lung cancer, age 50's Paternal great grandfather – lung cancer, age 60's
F	*PTEN* p.G165X	>100 adenomatous polyps CRC, age unknown Breast cancer, age unknown Age at testing 66	None reported
M	*CDH1* c.1565+1G>A	‘Few’ adenomatous colon polyps, age 54 Hyperplastic colorectal polyps CRC, age unknown	Mother – breast cancer, age 63 Sister – renal cell carcinoma, age 60s

CRC, colorectal cancer.

a Actionable is defined by the presence of general consensus guidelines for cancer prevention and/or early detection.

*Note*: Although the individuals in this table did not technically meet the syndrome-based testing or diagnostic criteria listed in Table1, a knowledgeable health care provider may have offered the testing in a number of these cases.

#### Description of patients with CHEK2 mutations

Table S1, Supporting Information summarizes information on the eight patients identified with *CHEK2* mutations. All of these individuals had a personal history of adenomatous polyps or CRC. None of these individuals had a personal history of breast cancer, but six had at least one family member with breast cancer.

## Discussion

To our knowledge, this study is among the first to report on a large clinical series of patients tested for inherited CRC through a NGS panel-based test. Among 586 patients tested, 42 had clinically actionable mutations, 8 had mutations in *CHEK2* (where clinical relevance remains uncertain), and 11 were *MUTYH* heterozygotes. Of 42 with clinically actionable mutations, at least 71% met national criteria for syndrome-based testing. The remaining 29% could not be definitively classified because of the possibility that additional clinical or family history could exist, but was not provided at the time of testing. As illustrated in Table3, a fair number of the unclassified patients had clinical histories suggestive of the condition identified through ColoNext™, whereas others appeared atypical. Among the atypical cases were two individuals in whom the panel-based testing identified a gene mutation in *SMAD4* indicating a diagnosis of juvenile polyposis; yet the polyp types reported on the TRF in one case did not include juvenile polyps and in the other case no history of polyps was reported. Additionally, there were four cases in whom mutations in polyposis genes were detected (i.e. two *MUTYH* and two *APC*); yet these individuals had insufficient numbers of polyps to meet NCCN testing criteria for polyposis syndromes. These findings highlight the potential for panel-based testing to identify mutations that might otherwise not have been identified because of limited medical or family history or an atypical presentation.

Of note, there were several cases where criteria for a specific genetic syndrome were clearly met in which there was an overlapping phenotype with other genetic conditions [e.g. CRC is associated with Lynch syndrome, but a similar phenotype may be observed with attenuated familial adenomatous polyposis (FAP)] 17. These cases represent situations where panel-based testing (compared to syndrome-based testing) may be particularly useful. Indeed, over half of the actionable mutations identified in this study occurred in genes associated with Lynch syndrome and several individuals with positive results had features of more than one condition.

As panel-based testing only became clinically available in March 2012, few reports exist to which our findings may be compared. One recent institution-based series of 50 patients with clinical panel-based testing for inherited cancer through Ambry included only five patients who received ColoNext™ testing 18. Of these, one had a *CHEK2* mutation and another was a *MUTYH* heterozygote.

Despite limited published data on clinical outcomes of panel-based testing, its potential benefits in the context of hereditary CRC have been reported 5,6. For example, panel-based testing can be less time consuming than syndrome-based testing 6. However, the cost-efficiency of panel *vs* syndrome-based testing will ultimately be determined by the relative costs of each, and costs continue to evolve within the current testing landscape. On the basis of our findings, although there are scenarios where panel-based testing may have been more cost-efficient, reality remains that syndrome-based testing would have been sufficient to identify the majority of patients with deleterious mutations. Consequently, the optimal and most cost-effective use of panel-based testing as a first-tier test *vs* a second-tier test (i.e. after syndrome-based testing is negative), remains to be determined. Although data suggest that several individuals in this study previously underwent syndrome-based testing, it is unknown whether these represent second-tier testing because initial testing could have been performed prior to the availability of panel-based testing. Furthermore, as information on tumor testing for Lynch Syndrome (either through IHC and/or MSI) was not included in the majority of TRFs, it was not possible for us to assess the efficacy of tumor testing to inform germline testing in our sample. Specifically, tumor testing (either through IHC and/or MSI) was mentioned in 21 cases. Of these, four had either abnormal IHC and/or MSI-H tumors, of which one had a mutation in MLH1 and the other three had prior testing for one or two Lynch syndrome genes. The remaining 17 cases for which this data was available through the TRF had normal IHC and/or MSI tumor testing.

Despite potential benefits, several anticipated challenges have been cited when conducting panel-based testing for inherited cancer predisposition 6,18. For example, testing for mutations in moderate penetrance genes could potentially lead to increased patient anxiety and/or excessive and unnecessary screening or preventive surgeries. Furthermore, there may be increased time for counseling when performing panel-based testing, in part because of the higher VUS rate and relaying information to patients about moderate penetrance genes. Nevertheless, as more individuals with mutations in moderate risk genes are identified, because of their inclusion on panel-based tests, additional information about their implications for cancer risk and medical management is expected to emerge.

The likelihood of detecting mutations in moderate penetrance genes is dependent on which of these genes is included on the specific panel-based test ordered. In our report focused on a colon-specific panel, *CHEK2* is the only moderate penetrance gene included within this panel. Different mutations in *CHEK2* may be associated with substantially different cancer risks 19. Clinical testing for *CHEK2* has been available for years, yet medical management of patients with *CHEK2* mutations is not well-defined; and cancer screening recommendations are primarily based on patients' personal and family medical histories. Individuals with *CHEK2* mutations in this study may have a higher incidence of CRC because of selection bias. However, prior to the introduction of panel-based testing, clinical testing for *CHEK2* mutations was rare, and our results may reflect that these mutations are more common than previously believed. While most cancers in the eight *CHEK2* families were later onset, seven of them had at least one individual with a cancer diagnosed before the age of 50. In these cases it may be appropriate to consider lowering the age they begin cancer screening for the affected organ 19.

Additional challenges can occur related to *MUTYH* associated polyposis (MAP) which confers up to 63% CRC risk by age 60 20. Unlike other hereditary CRC syndromes that are included on ColoNext™ testing, MAP is an autosomal recessive condition; therefore, parents and children of individuals with MAP are rarely affected. Nevertheless, the question of whether monoallelic *MUTYH* mutation carriers have a moderately increased CRC risk remains unclear 20–22.

Another factor to consider with expansion of panel-based testing is an expected increase in the rate of VUS results. This study found a relatively high VUS rate, occurring in 20% of the individuals tested. Interestingly, nearly half of all VUS results in the current study occurred in four Lynch syndrome genes which comprised less than a third of the 14 ColoNext™ genes, which translates to a VUS rate of ∼10% consistent with reports from other laboratories that perform LS genetic testing 23,24. Although VUS results remain an important concern, it is anticipated that the ability to classify VUS results will improve with more widespread testing and data sharing among researchers and laboratories (including Ambry).

Study strengths include the large sample size from the first commercial laboratory to offer clinical panel-based testing for inherited cancer predisposition. Furthermore, generalizability of findings is enhanced given that our sample included a diverse group of patients from over 200 US institutions. Despite these strengths, our sample encompassed early adopters of a single cancer panel primarily ordered by genetics professionals. Therefore, mutation and VUS rates will vary across panels and may change over time as panel-based tests are more widely diffused. Furthermore, our reliance on information in the TRF without medical record and family history verification as well as lack of information about tumor testing preceding germline testing in most cases (to identify possible Lynch Syndrome) are inherent limitations; however this approach also enabled the large sample size for the study. Moreover, information contained in the TRF was sufficient to suggest that phenotypes associated with mutations in certain genes and criteria for testing may need to be expanded as more panel-based testing identifies other atypical cases.

As adoption of panel-based testing for hereditary cancer syndromes continues to spread it is important to ensure that patients receive accurate information about their test results, particularly for the substantial number of patients who will receive inconclusive results as well as those found to have mutations in rarer inherited cancer genes and moderate penetrance genes. This study suggests that genetics professionals comprise most of the early adopters of panel-based testing. However, it is anticipated that over time, the use of NGS panels will diffuse to providers with less specialized training in genetics. Educational efforts, preferably spearheaded by academic institutions or professional organizations rather than by commercial laboratories, will therefore be needed as many providers in the US have been shown to lack knowledge of fundamental genetic concepts and VUS results 25–27. Furthermore, although panel-based testing may reduce the need to perform a differential diagnosis up front, clinicians who order testing must still be knowledgeable about clinical diagnostic criteria for hereditary cancer syndromes and management recommendations in order to provide the most appropriate application of test results to patient care. Finally, it remains uncertain whether identification of moderate penetrance genes truly helps guide cancer screening decisions over and above what would be recommended based on comprehensive collection of family history without conducting testing.

Ultimately, to help prevent negative outcomes, it is imperative that additional guidelines regarding the use of panel-based testing be developed. The current 2013 ‘NCCN guidelines for Colorectal Screening’ do not mention panel-based testing. Additional research is needed to update these practice guidelines so they may better address the unique practice-based issues and advantages of panel-based testing. Uncertainties associated with moderate penetrant genes, such as *CHEK2*, highlight the importance of research studies and academic registries. In addition, research is needed to identify the optimal counseling approach for panel-based testing.

Overall, this study provides a broader picture of panel-based testing for hereditary CRC than previously available and suggests that testing may: (1) cast a wider net, and in some cases identify mutations in genes that might not otherwise be tested because of an atypical phenotype and (2) be an efficient approach when patients present with features of more than one hereditary CRC syndrome. Despite highlighting these potential benefits of panel-based testing, this study raises additional questions and concerns that will need to be addressed through clinical research and education.

## Supporting Information

The following Supporting information is available for this article

Table S1 Descriptions of individuals with a pathogenic mutation in *CHEK2* only.
