# Supplementary material for: Panel-based testing for inherited colorectal cancer: a descriptive study of clinical testing performed by a US laboratory
Source: Clin Genet. 2014 Mar 20;86(6):510–20. doi: 10.1111/cge.12359 (PMC4127163; doi:10.1111/cge.12359)
Supplement: Table S1 — Descriptions of individuals with a pathogenic mutation in CHEK2 only. [file cge0086-0510-SD1.doc]

Supplemental Table A. Descriptions of individuals with a pathogenic mutation in *CHEK2* only

| **Gender** | **Mutation** | **Personal history** | **Family history** |
| --- | --- | --- | --- |
| M | c.1100delC | 10-19 adenomatous polyps, age 50  Papillary thyroid cancer, age 45 | Sister- CRC, age 60  Father- CRC, age 59  Uncle- CRC, ages 59 and 62  Family history ovarian cancer (not otherwise specified) |
| F | p.S428F | 2-5 adenomatous polyps, age 39 | Maternal grandfather- CRC, age 75  Maternal grandmother- CRC, age85  Paternal aunt- CRC, age 45  Paternal cousin- adenomatous polyps, age 45  Paternal great aunt- breast cancer, age unknown |
| M | p.R95X | 10-19 adenomatous polyps, age 50  CRC, age 50 | Two paternal first cousins- breast cancer, ages 50's  Paternal aunt- breast cancer, age 50's  Paternal uncle- CRC, age 55  Father- lymphoma, age 60's |
| F | c.1100delC | CRC, age 41 | Paternal aunt – breast cancer, age 41 |
| F | p.I157T | CRC, age 72 | Mother- breast cancer, age70's  Sister- breast cancer, age 50; thyroid cancer age 30; uterine cancer age 60’s  Sister- breast cancer age 40’s  Brother-CRC, age 49  Maternal niece- thyroid cancer, age 30’s  Son- colon polyps, age 40's |
| M | c.1100delC | CRC, age 53 | Sister- CRC, age 48  Brother- melanoma, age 56  Father- colorectal, age 54  Paternal grandfather – CRC, age unknown  Paternal 3^rd^ cousin – CRC, age 54  Maternal uncle – bladder cancer, age 65  Maternal cousin – bladder cancer, age 55  Maternal grandmother – breast cancer, age 85 |

Supplemental Table A continued.

| **Gender** | **Mutation** | **Personal history** | **Family history** |
| --- | --- | --- | --- |
| F | p.I157T | 2-5 Adenomatous polyps, ages 20 and 52 | Mother- CRC, age 27; polyps age 20’s; thyroid cancer age 30’s  Father-CRC, age 57  Brother- 2-5 polyps, age 50’s  Paternal Grandfather- CRC, age 60's  Maternal Grandmother- CRC, age 55  Maternal uncle- 2-5 polyps, age 50’s |
| M | c.1100delC | Colorectal cancer, age 39 | Mother- breast cancer, age 74  maternal grandmother- uterine cancer, age 64  maternal great grandmother-breast cancer, age unknown  Father- colorectal cancer, age 81; prostate cancer, age 76; bladder cancer, age 77 |

CRC=colorectal cancer
